# A new species of *Nilobezzia* Kieffer (Diptera, Ceratopogonidae) from the mangrove forest of Hainan Island, China

**DOI:** 10.3897/zookeys.893.39032

**Published:** 2019-12-02

**Authors:** Chunqiao Li, Glenn Bellis, Xiaoxiang Wu, Jiahui Li

**Affiliations:** 1 Key Laboratory of Green Prevention and Control of Tropical Plant Diseases and Pests, Ministry of Education, College of Plant Protection, Hainan University, Haikou, 570228, China Hainan University Haikou China; 2 Research Institute for the Environment and Livelihoods, Charles Darwin University, Darwin, Northern Territory, Australia Charles Darwin University Darwin Australia

**Keywords:** biting midges, China, *Nilobezzia bamenwana*, predaceous midges

## Abstract

A new species of *Nilobezzia* Kieffer, *Nilobezzia bamenwana* Li & Li, **sp. nov.**, collected from Bamenwan mangrove forest of Hainan Island, China, is described and illustrated based on female adults. The genus was previously known to have a single species occurring on the island.

## Introduction

*Nilobezzia* Kieffer, 1921 is a genus of predaceous ceratopogonid midges in the tribe Johannsenomyiini of the subfamily Ceratopogoninae. It is striking that adult females prey on males while mating (Downes 1969). There are 74 species worldwide (Borkent 2016), of which ten species are distributed in mainland China and Taiwan (Yu et al. 2005). Only one species, *Nilobezzia duodenalis* Liu, Yan & Liu, has been reported from Hainan Island, which was collected from Limu Mountain in the central area of the island (Liu et al. 1996; Wang et al. 2011). The aim of this contribution is to describe a new species of *Nilobezzia* which was discovered as part of an ongoing investigation of the Ceratopogonidae of Hainan Island.

## Materials and methods

Specimens were collected with a light trap from Bamenwan mangrove forest near Wenchang, Hainan Province, China. The holotype and two paratypes were processed for DNA barcoding of the mitochondrial 5’ cytochrome coxidase I gene region, and subsequently mounted onto microscope slides following non-destructive tissue digestion as described by Bellis et al. (2013). DNA barcode sequences compliant with quality assurance criteria of the Barcode of Life Data Systems (BOLD) database (Ratnasingham and Hebert 2007) were submitted with associated specimen details as a dataset (http://doi.org/10.5883/DS-NILO). DNA barcode sequences were submitted to GenBank (accession numbers MN135243–MN135245).

Details of colour were taken from specimens kept in ethanol. Measurements of the holotype are provided with the range of variation of the paratypes presented in parentheses. The terminology follows Debenham (1974) and Wirth and Ratanaworabhan (1981). All type specimens are deposited in the Institute of Tropical Agriculture and Forestry, Hainan University, China.

## Taxonomy

### Nilobezzia

Taxon classificationAnimaliaDipteraCeratopogonidae

Kieffer, 1921

A46C75DB-CE6C-5A76-B034-59AE587991DD

Nilobezzia Kieffer, 1921: 24. Type species: Nilobezzia armata Kieffer, 1921 by monotypy.
Parrotia Kieffer, 1923: 140. Type species: Parrotia flaviventris Kieffer, by original designation. Synonymised by Wirth et al. 1974: 603.
Crespinia Kieffer, 1923: 141. Type species: Crespinia brevipalpis Kieffer by monotypy. Synonymised by Wirth et al. 1974: 603.
Sphaerobezzia Zilahi-Sebess, 1940: 108 (as subgenus of Bezzia). Type species: Bezzia paradoxa Zilahi-Sebess, by monotypy. Synonymised by Wirth et al. 1974: 603.

### Nilobezzia bamenwana

Taxon classificationAnimaliaDipteraCeratopogonidae

Li & Li sp. nov.

C511A14E-5AC0-59AA-812D-223629A70852

http://zoobank.org/8E69FC30-F2CC-428E-A9D5-AB6E401833AE

Figures 1–11 , 12–16

#### Type material.

***Holotype*.** CHINA • ♀, slide, Hainan Province, Wenchang City, Bamenwan mangrove forest; 19°37'38"N, 110°47'10"E, 18 Jun 2018; Chunqiao Li leg., light trap; cer250-1, GenBank MN135245.

***Paratypes*.** CHINA • 9♀, same data as holotype, six paratypes (cer250-2–cer250-7) mounted on slides, three kept in ethanol (cer250-8–cer250-10). GenBank MN135243 and MN135244.

#### Diagnosis.

The only species of *Nilobezzia* with the following combination of characters: body longer than 3.5 mm; femora and tibiae with spines scattered along their length; femora distinctly yellow basally and dark brown distally, tibiae dark brown with subapical pale bands; wing with a single radial cell and spermathecae unequal in size and without necks.

#### Description.

**Figures 1–11.**
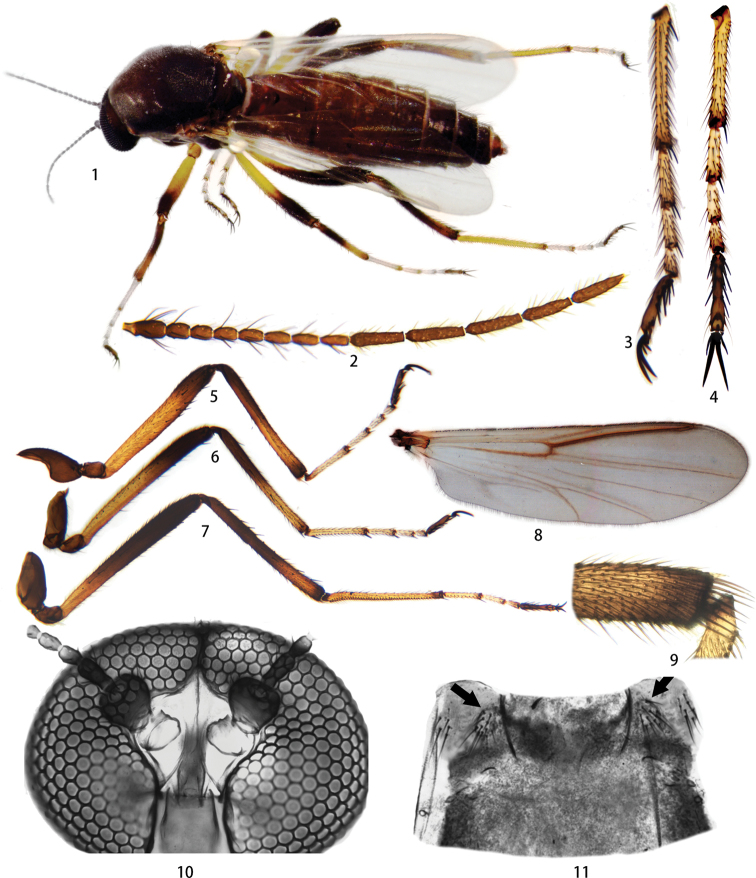
Female of *Nilobezzia bamenwana* sp. nov. **1** habitus in dorsal view **2** antenna **3** midtarsus, anterior view **4** foretarsus, ventral view **5** foreleg **6** midleg **7** hind leg **8** wing **9** hind tibial comb **10** head, dorsal view **11** abdominal tergite I, dorsal view.

**Female. *Body*** (Fig. 1) 4.25 (3.85–4.28) mm in length. Wing 2.69 (2.69–2.95) mm in length.

***Head dark brown*.** Eyes contiguous, bare (Fig. 10). Antenna dark brown with slightly paler pedicel, basal flagellomeres short and stout, distal 5 flagellomeres each much longer than basal 8 flagellomeres, lengths in ratio of 96:47:47:46:51:50:58:64:129:123:127:111:140; AR 1.37 (1.17–1.37, *N* = 5) (Fig. 2). Maxillary palpi brown, 5-segmented, third segment long, not distinctly swollen, a few scattered hyaline sensillae preapically, lengths in ratio of 17:33:86:43:42, PR 3.07 (2.99–3.44) (Figs 14, 16). Mandible with seven coarse teeth (Figs 14, 15).

***Thorax*.** Scutum dark brown, some specimens with humeral area slightly paler, with fine microsetae and several bristles near base of wings. Scutellum and postscutellum concolourous with scutum. Coxae dark brown, trochanters light brown; forefemur, midfemur with basal 2/3 yellow and distal 1/3 dark brown, basal 1/3 of hind femur yellow, distal 2/3 dark brown; basal 1/2 of fore tibia and mid tibia dark brown, distal 1/2 yellow, except narrow dark apex; 2/3 of hind tibia dark brown, apical 1/3 slightly paler, dark apex (Figs 1, 5–7). All femora and tibiae with many scattered black spines over their entire length, variable in number; hind tibial comb (Fig. 9) with nine spines. Tarsomeres I–IV yellow except brown apices; tarsomere V entirely dark brown; foretarsomere and hindtarsomere I–II and midtarsomere III each with single apical spine, midtarsomere I–II with two apical spines, midtarsomere I also with a basal spine, foretarsomere and hind tarsomere III and tarsomere IV of all legs without apical spine, tarsomere V (Fig. 4) with 14 ventral batonnets; claws equal, 0.8 times the length of tarsomere V and bearing two strong basal teeth on the outer surface ca. 0.3 times of length of claw (Figs 3, 4). RL-L 1013:948:377:196:142:80:245 and TR 1.92 (1.78–2.08, *N* = 5) in fore leg, RL-L 1460:1080:533:231:173:91:225 and TR 2.30 (2.15–2.56, *N* = 5) in mid leg, RL-L 1508:1269:1000:320:236:133:240 and TR 3.10 (2.90–3.10, *N* = 5) in hind leg. Wing membrane pale grey, CR 0.82 (0.81–0.82, *N* = 5), a single radial cell (Fig. 8). Haltere white.

**Figures 12–16.**
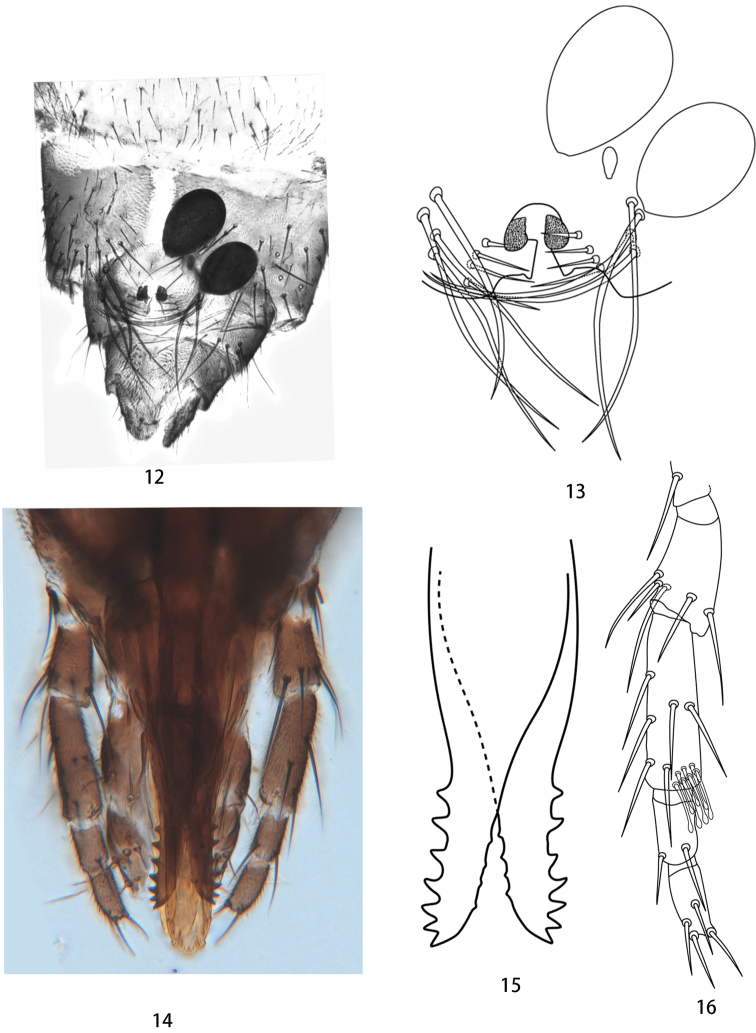
Female of *Nilobezzia bamenwana* sp. nov. **12** terminal four segments of abdomen, ventral view **13** spermathecae and hair tufts, ventral view **14** proboscis and palpi, anterior view **15** mandibular teeth, anterior view **16** maxillary palpus, anterior view.

Abdominal tergites brown, tergite I (Fig. 11) with lateral tufts of 10–12 short setae arranged in oval area, with anteromesal dark, triangular spot; abdominal segment VIII with a pair of subquadrate genital sclerotations near semi-circular gonopore, ventral hair tufts dark and conspicuous, each comprising a row of four or five long black bristles and a row of four short bristles on each side (Figs 12, 13); cerci brown; two dark brown spermathecae, large, oval, unequal, 129×83 (109–129×67–83) μm and 94×66 (90–110×57–66) μm, neck absent, and a third vestigial spermatheca present (Figs 12, 13).

**Male.** Unknown.

#### Etymology.

The name *bamenwana* refers to the collecting location of the species.

#### Distribution.

Known only from the Bamenwan mangrove forest of Hainan Prov., China.

#### Discussion.

Female specimens of *Nilobezzia bamenwana* run to genus *Nilobezzia* in the key of Wirth et al. (1974) and conforms to the diagnosis of *Nilobezzia* provided by Debenham (1974) and Wirth and Ratanaworabhan (1981). The only other species of *Nilobezzia* recorded from mangrove forest is *N. virago* Debenham which was recorded from many different habitats including a single female specimen collected from mangroves in Australia (Debenham 1974).

*Nilobezzia bamenwana* runs to *N. acanthopus* (de Meijere) in the key to Southeast Asian species by Wirth and Ratanaworabhan (1981) but in the latter species the forefemur and midfemur are entirely yellowish and the spermathecae are equal in size. India has 18 described species of *Nilobezzia* (Mazumdar et al. 2009), some of which possess similar leg colour patterns and other characteristics to *N. bamenwana*, but with wings shorter than 2.5 mm and significantly smaller than *N. bamenwana* and none appear to be associated with mangroves. *Nilobezzia bamenwana* runs to *N. opaca* Das Gupta in the key by Mazumdar et al. (2009), but that species can be distinguished by the obvious neck of spermathecae and legs excepting the midfemora, entirely brown. It runs to *N. formosana* (Kieffer) in the key to Chinese species of *Nilobezzia* by Yu et al. (2005), but that species is much smaller in size with a wing length of only 2.11 mm. *Nilobezzia bamenwana* is allied to *N. japana* Tokunaga in general colour, but the latter species is smaller (body length 3.2 mm), has more extensive dark markings on the forefemur and midfemur, and the foretibia lacks spines.

## Supplementary Material

XML Treatment for Nilobezzia

XML Treatment for Nilobezzia bamenwana
